# The influence of spatial pattern on visual short-term memory for contrast

**DOI:** 10.3758/s13414-014-0671-x

**Published:** 2014-04-09

**Authors:** Yue Xing, Tim Ledgeway, Paul McGraw, Denis Schluppeck

**Affiliations:** Visual Neuroscience Group, School of Psychology, University of Nottingham, Nottingham, NG7 2RD UK

**Keywords:** Keyword, Memory, Visual working memory, Short-term memory, Spatial vision, Visual perception, Contrast

## Abstract

Several psychophysical studies of visual short-term memory (VSTM) have shown high-fidelity storage capacity for many properties of visual stimuli. On judgments of the spatial frequency of gratings, for example, discrimination performance does not decrease significantly, even for memory intervals of up to 30 s. For other properties, such as stimulus orientation and contrast, however, such “perfect storage” behavior is not found, although the reasons for this difference remain unresolved. Here, we report two experiments in which we investigated the nature of the representation of stimulus *contrast* in VSTM using spatially complex, two-dimensional random-noise stimuli. We addressed whether information about contrast per se is retained during the memory interval by using a test stimulus with the same spatial structure but either the same or the opposite local contrast polarity, with respect to the comparison (i.e., remembered) stimulus. We found that discrimination thresholds got steadily worse with increasing duration of the memory interval. Furthermore, performance was better when the test and comparison stimuli had the *same* local contrast polarity than when they were contrast-reversed. Finally, when a noise mask was introduced during the memory interval, its disruptive effect was maximal when the spatial configuration of its constituent elements was uncorrelated with those of the comparison and test stimuli. These results suggest that VSTM for contrast is closely tied to the spatial configuration of stimuli and is not transformed into a more abstract representation.

Several previous studies have shown that judgments on some properties of visual stimuli, such as the spatial frequency of sinusoidal gratings, are barely affected by relatively long delays between the two stimuli that need to be compared. Discrimination performance in a two-interval forced choice task, for example, does not decrease significantly even for memory intervals of 30 s (Magnussen & Greenlee, 1992). For other basic image properties such as the contrast of gratings (Lee & Harris, 1996; Magnussen, Greenlee, & Thomas, 1996) and orientation of single bars (Magnussen & Greenlee, 1999; Magnussen, Landrø, & Johnsen, 1985; Vogels & Orban, 1986); however, such “perfect storage” behavior is not found (for a review, see also Pasternak & Greenlee, 2005).

The differential characteristics of visual short-term memory (VSTM) for various visual features are consistent with the view that they are processed in parallel and to some extent independently from each other, allowing for limited interaction across domains (Magnussen et al., 1996). Moreover, electrophysiology and neuroimaging experiments have found sustained activity in early visual cortex during the memory interval, suggesting that neurons in these areas are recruited for perceptual maintenance in VSTM tasks (see, e.g., Bisley, Zaksas, Droll, & Pasternak, 2004; Zaksas & Pasternak, 2006). These findings were interpreted in support of the “sensory recruitment hypothesis”—the idea that the same cortical areas and neural circuitry are used during the processing of visual stimuli for perception as well as their maintenance in VSTM. Also, a growing body of functional magnetic resonance imaging (fMRI) studies now suggest a role for early visual areas in mediating VSTM (Ester, Serences, & Awh, 2009; Harrison & Tong, 2009; Sneve, Alnæs, Endestad, Greenlee, & Magnussen, 2012).

However, most of the previous experiments examining the sensory-recruitment hypothesis have focused on features of simple visual stimuli, such as spatial frequency and orientation, and much less is known about the characteristics of VSTM for stimulus *contrast*. The neural representation for spatial frequency or orientation in early visual cortex relies on subpopulations of neurons with specific tuning preferences (for a review, see, e.g., De Valois & De Valois, 1988). For stimulus contrast, on the other hand, the activity of most visual cortex neurons shows a monotonic relationship with increasing contrast (Albrecht & Hamilton, 1982). Additionally, unlike with stimulus orientation, there is no known columnar organization for neurons with similar contrast-response functions. The representation of these stimulus properties is therefore fundamentally different.

VSTM for basic stimulus properties has been reported to be both *dimension-selective* (Blake, Cepeda, & Hiris, 1997; Magnussen & Greenlee, 1992) and *feature-selective* to memory interference (Magnussen & Greenlee, 1992). Memory masking of spatial frequency, for example, is selective along the dimension of the task-relevant visual information (spatial frequency), but independent from other visual dimensions (e.g., orientation). Recent studies looking at memory for color and motion stimuli have further elucidated this masking, and their results tie in well with those presented here (Nemes, Parry, Whitaker, & McKeefry, 2012; Pavan, Langgartner, & Greenlee, 2013). However, unlike with spatial frequency or orientation, the neuronal representation of contrast is not narrowly tuned, so a question remains open: What kinds of masks lead to larger disruption of performance when they are delivered during the memory interval—masks that are similar (or the *same*) in contrast, implying feature selectivity, or masks that are *different* in contrast?

Here, we investigated how perceptual information about stimulus contrast is encoded and maintained in memory.

In the first experiment, we tested the effects of delay duration on contrast VSTM performance using two-dimensional (2-D) noise patterns. The results from this experiment showed that the contrast is not perfectly stored over extended durations. Three causes may lead to the loss of contrast memory with increasing time between the comparison and test stimuli: (1) Contrast information decays over time. (2) The neuronal representation of contrast becomes increasingly noisy, even though contrast storage is still intact. (3) Different contrast levels converge onto an average contrast. Lee and Harris (1996) looked at possible reasons for contrast decay. They performed a delayed contrast discrimination task similar to ours, but measured the point of subjective equality (PSE) and found no change with increasing delay, suggesting that the memory representation of contrast becomes noisier over time, leading to higher discrimination thresholds, rather than being the product of a systematic bias in the remembered contrast.

In Experiment 2, we examined the effect of a mask during the memory interval on contrast judgments of noise patterns. Our results suggest that the memory representation for stimulus contrast is closely tied to the spatial pattern that is being remembered, and that it is not abstracted from the low-level visual representation.

## General method

### Participants

Four observers, including two experienced observers and two naïve participants, consented to participate in these experiments. In a pilot experiment, we also tested two additional participants to set the range of parameters used in the experiments presented here. The procedures were approved by the Medical School Research Ethics Committee at the University of Nottingham. All of the participants had normal or corrected-to-normal vision.

### Apparatus

Stimuli were generated on a Macintosh computer using custom software written for the stimulus generation tools MGL^1^ in MATLAB (MathWorks, Natick, MA). A CRT monitor (resolution 1,024 × 768 pixels, refresh rate 85 Hz, mean luminance 45.5 cd/m^2^) placed at a viewing distance of 57 cm was used to display the visual patterns. The monitor’s gamma nonlinearity was corrected and checked using a sensitive psychophysical approach (Ledgeway & Smith, 1994; Lu & Sperling, 2001).

## Experiment 1: effect of memory duration on VSTM for stimulus contrast of complex spatial patterns

**Fig. 1:**
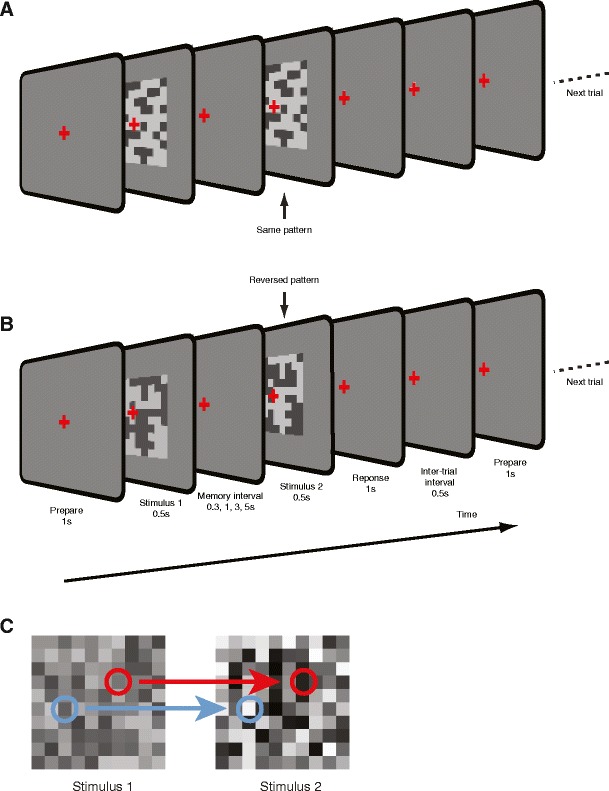
VSTM task for the contrast of random binary noise patterns. The comparison stimulus (Stimulus 1) and the test stimulus (Stimulus 2) were separated by a memory interval of either 0.3, 1, 3, or 5 s. At the end of each trial, participants had to respond by pressing a button to indicate which stimulus had the higher contrast. (**a**) Noise patterns in both stimulus intervals were kept the same, or (**b**) the noise patterns in the two stimulus intervals were contrast-inverted. Subsequent trials started after a 1-s intertrial interval. (**c**) Control stimuli. Luminance levels of the individual elements of these stimuli were sampled from a uniform probability distribution with a broad range of grayscale levels (rather than just the two used in the binary stimuli) and were spatially uncorrelated between the two intervals. Here, Stimulus 1 has lower contrast than Stimulus 2, but participants could not use the luminance of individual elements to make a reliable judgment, since some elements became darker (top circles and arrow) and some brighter (lower circles and arrow). Participants had to extract and utilize the overall contrast of the patterns to perform the task

The purpose of this experiment was to characterize VSTM for the contrast of spatially complex 2-D stimuli. The aim was to examine whether the contrast signal is extracted independently of the local arrangement of luminance elements. Two experimental conditions were designed for this purpose. In one condition (referred to as the “identical-pattern” condition and shown in Fig. 1a), the same noise pattern (at different contrasts) was displayed in the two stimulus intervals of a trial. In another condition (“reversed-pattern” condition, shown in Fig. 1b), the luminance distributions of the stimuli were reversed, such that formerly white elements were black, and vice versa. Therefore, the same contrast energy was maintained across the conditions, but the spatial patterns and the polarity of local edges were reversed in each of the trials in the second condition.

If the performance in the contrast discrimination task were independent of the distribution of the luminance elements that conveyed the contrast, then the thresholds of these two conditions would be similar, regardless of the stimulus profile. Alternatively, if observers encode and rely on spatially local contrast information to perform the task, we would expect differences in performance across these two conditions.

### Method

#### Visual stimuli and procedure

A schematic representation of the stimuli and procedure is shown in Fig. 1. Each trial consisted of two 500-ms stimulus intervals separated by a memory interval (either 0.3, 1, 3, or 5 s) during which a mean luminance gray screen, with a fixation cross, was displayed. One of the two stimulus intervals contained a lower-contrast stimulus (pedestal), and we added a signal randomly chosen from seven different contrast increments to this pedestal for the other stimulus interval. Observers were asked to fixate a central cross throughout each block. At the end of each trial, observers were required to choose the interval containing the higher-contrast pattern. Feedback (a color change of the fixation cross) was given to indicate whether the answer was correct (green) or incorrect (red). Each trial was separated by a 1,000-ms intertrial interval. Each run contained 40 discrimination trials per point on the psychometric curves.

For each run, 20 images composed of a 10 × 10 array of square elements (20° × 20° visual angle in total) were drawn and stored. Approximately half of the 100 square elements (randomly chosen if a uniform random number exceeded 0.5) were assigned to be “black,” and the rest were “white” (see Fig. 1). We used 20 exemplars of noise stimuli to ensure that participants could not learn a specific spatial cue in the pixel noise patterns across trials to perform the task.

We note that stochastic patterns created in this way, in effect, may not be perfectly balanced, because they are unlikely to contain exactly equal numbers of elements of each luminance polarity. Additionally, participants could potentially base their judgments on the absolute luminance levels assigned to individual elements of the binary noise stimuli, rather than on the image contrast (i.e., the luminance difference between elements with opposite polarity). To control for these possible confounds, we performed two additional sets of tests on four participants to confirm that the participants (a) based their judgments on overall estimates of contrast and (b) were not biased by small imbalances of “white” and “black” elements (data are not shown).

After piloting the noise stimuli with pedestals at three Michelson contrasts (20 %, 40 %, and 70 %) on two observers and establishing that the results were consistent across the different pedestals, in order to maximize the number of trials, we then reduced the set of stimuli to only one pedestal (70 % Michelson contrast). All observers performed the same procedure as in the piloting session, but with four different memory interval durations (0.3, 1, 3, and 5 s).

#### Analysis: determination of thresholds

We used nonlinear regression to fit sigmoid (Weibull) functions to the psychophysical data (psignifit toolbox, version 2.5.6 for MATLAB; http://bootstrap-software.org/psignifit) for each participant and memory interval. From each fit, the contrast increment necessary for participants to achieve 75 % correct performance was estimated. Each psychometric curve was based on data from 280 trials (40 repeats at each of seven stimulus levels).

### Results

**Fig. 2:**
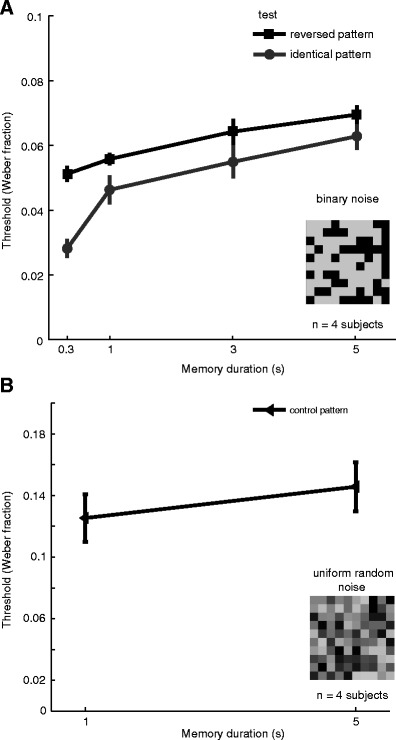
(**a**) Discrimination thresholds (Weber fractions) for the contrasts of random noise patterns as a function of memory interval in the VSTM task. The light gray line indicates thresholds for trials in the identical-pattern condition, when the two stimuli had the *same* spatial pattern. The black line indicates thresholds for trials in the reversed-pattern condition, when the two stimuli were contrast-reversed. Error bars indicate *SEM*s across (*n* = 4) participants. (**b**) Discrimination thresholds (Weber fractions) in the control experiment using stimuli created with uniform random, rather than binary, noise. With such stimuli, it is impossible to perform the task reliably by basing judgments on the apparent brightness of individual elements within the display; observers must use the contrast across the image. Observers showed the same drop in performance with retention interval as when binary noise patterns were used. Error bars indicate *SEM*s across (*n* = 4) participants

We found that thresholds increased with memory interval duration in both cases: when judgments had to be made on identical patterns, as well as when they had to be made on contrast-reversed patterns. Participants were clearly able to perform the task and to discriminate the contrast changes between the comparison and test stimuli, but they showed elevated thresholds with increasing memory durations (Fig. 2a). Interestingly, the Weber fractions for the reversed-pattern condition were consistently higher than those for the identical-pattern condition by about ~17 %, indicating an additional cost to performance when the patterns in the task were simply contrast-reversed versions of each other. To assess the statistical significance of the changes in threshold, we conducted a two-way repeated measures analysis of variance (ANOVA) on retention duration (0.3, 1, 3, and 5 s) and stimulus pattern type (identical and reversed patterns). The main effects of both memory interval duration and stimulus type were both significant [*F*(3, 31) = 19.49, *p* < 10^−8^, and *F*(1, 31) = 21.94, *p* < .001, respectively]. The interaction was not significant [*F*(3, 31) = 2.03, *p* = .1366]. This result suggests that the visual system may encode and represent the spatial configuration of the stimulus to facilitate the process of VSTM for contrast.

### Control: participants used contrast information to perform the task

To eliminate the possibility that observers were simply relying on spatially localized *luminance* cues in the binary noise images to perform the task, we also repeated a subset of conditions using noise images in which the luminance levels of the individual elements were sampled from a uniform distribution with a broad range of possible gray levels (rather than just two; see Fig. 1c). Furthermore, we measured performance when the comparison and test stimuli had different (uncorrelated) samples of this noise. With such stimuli it is impossible to perform the task reliably by basing judgments on the apparent brightness of individual elements within the display; observers must use the contrast across the image. Under these conditions, we found that observers were able to perform the discrimination task well, and performance showed the same drop in performance with retention interval as was found using binary noise patterns (Fig. 2b). The *overall* increase in thresholds was likely associated with the contrast reduction between stimuli produced from binary and uniform random noise (i.e., the root-mean-square contrast of uniform noise patterns is much lower than that of equivalent binary noise, even if the patterns have the *same* overall Michelson contrast; Bex & Makous, 2002).

## Experiment 2: disrupting VSTM for contrast with complex spatial masks

Experiment 1 demonstrated that the memory for image contrast decays over time. Moreover, the marked difference between the performance found for the identical-pattern and the reversed-pattern conditions suggests that the information about stimulus contrast that has to be encoded and maintained for a discrimination may be closely related to a pictorial representation of the pattern. In Experiment 2, we investigated this further and tried to disrupt the contrast memory representation by using a “memory mask.” Rather than being presented simultaneously during the stimulus presentation, the masks here were well outside the time window in which traditional masking could affect the initial perception or encoding of the comparison or test stimulus (Breitmeyer, 1984). We presented the same kind of noise patterns (as our comparison and test stimuli) halfway through the memory interval in each trial. Furthermore, we tested the effect of two factors when presenting the masks: (1) the spatial pattern of the noise mask could be either “identical” to or “different” (i.e., composed of a new stochastic sample of elements) from the comparison and test stimuli, and (2) the contrast of the masks could be either lower than, the same as (on average), or higher than the comparison and test stimuli.

Considering the first manipulation: If VSTM for contrast is partially held in sensory visual areas, then the viewing and encoding features of an additional stimulus pattern with a different spatial/contrast layout should interfere with memory performance. This hypothesis is based on the assumption that processing another visual stimulus (contrast) would interfere with the ongoing memory process occurring in shared functional areas. Moreover, if a pictorial representation for a particular stimulus is indeed tied to the activity of a particular subset of neurons, then a mask image that is substantially different should exert a more disruptive effect than a masking pattern that is identical, because it will likely involve the activity of a different subset of neurons in sensory areas.

Considering the second manipulation (the effect of mask contrast on thresholds): Because, unlike with spatial frequency or orientation, the neuronal representation of contrast is not narrowly tuned, there is an open question: Do masks at the *same* or *different* contrast produce a larger disruption of performance when they are delivered during the memory interval? One possibility is that the disruption would be largest for the highest-contrast masks, and another is that disruption would be largest if the mask contrast was closest to that of the comparison/test stimuli (see also McKeefry, Burton, & Vakrou, 2007).

### Method

#### Visual stimuli and procedure

**Fig. 3:**
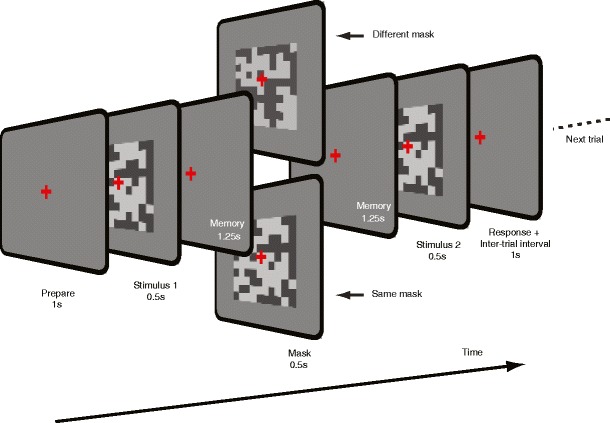
VSTM task for the contrast of random noise patterns with masking. On each trial, two stimuli of different contrasts were presented for 0.5 s each, separated by a 3-s memory interval. During the middle of the memory interval, a mask stimulus was presented. As before, participants had to indicate by pressing a button whether the comparison stimulus (Stimulus 1) or the test stimulus (Stimulus 2) had higher contrast. The bottom mask stimulus has the same spatial noise pattern as in the stimulus intervals, and the top mask has a different (uncorrelated) binary pixel noise pattern. The next trial started after an intertrial interval of 1 s

The stimuli and procedure were similar to those in Experiment 1, with the following modifications: We introduced masks (noise patterns) midway through the 3-s memory interval (Fig. 3). The mask lasted as long as the stimulus interval (500 ms), and we used the same procedure for generating the random pattern masks as for the comparison and test stimuli. Across a series of trials, we manipulated the layout of the mask (either an identical or a different pattern) and the contrast of the mask (either equal to the mean of the comparison and test stimuli, 10 % higher than the maximum contrast, or 10 % lower than the minimum contrast). At the end of each trial, feedback was given. Six different blocks of these mask contrast conditions were interleaved. In total, at least 40 trials were presented for each point on the psychometric curve when measuring discrimination thresholds for each participant.

### Results

**Fig. 4:**
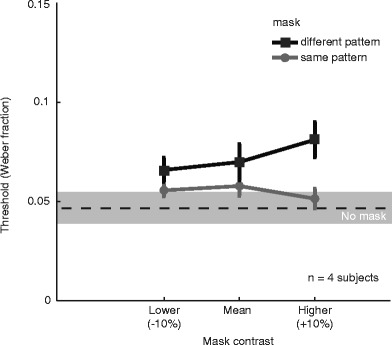
Effect of masks on discrimination thresholds (Weber fractions) in the VSTM task. The gray line and symbols show the effect of masking on thresholds when the spatial pattern of the stimuli and mask were the same, whereas the black line and symbols show thresholds when the masks were different from the contrast stimuli (1 and 2). Error bars indicate *SEM*s across (*n* = 4) participants. The dashed line and shaded area indicate the threshold and ±1 *SEM*, respectively, across the same group of participants when no mask stimulus was presented

We found that introducing a mask during the memory interval had a deleterious effect on thresholds. The data presented in Fig. 4 show discrimination thresholds as a function of the mask contrast for trials in which it had the same spatial pattern as (gray symbols and line) or a different spatial pattern from (black symbols and line) the comparison and test stimuli. As compared with the no-mask condition, which is indicated by the horizontal dashed line, with ±1 *SEM* across observers in the shaded area, it is clear that the overall performance deteriorated when a noise mask was present. This disruptive effect was more pronounced when the mask and stimulus patterns were different.

The relative contrast of the mask had little effect on performance. We manipulated mask contrast with three different values (–10 %, mean, and +10 % of the range of contrasts). On inspection, only the condition with the higher-contrast mask and a different pattern appears to show a clear elevation of threshold. To test statistical significance, we performed a two-way repeated measures ANOVA on changes in the discrimination thresholds for mask contrast (low, mean and high) and mask pattern type (identical and different). The presence of a particular masking pattern had a significant effect on discrimination performance [*F*(1, 23) = 10.58, *p* < .01], whereas mask contrast had no statistically significant effect [*F*(2, 23) = 0.36, *p* = .70, n.s.]. We found no significant interaction between mask pattern type and mask contrast [*F*(2, 23) = 1.35, *p* = .28].

## Discussion

The results from both experiments suggest that the mechanisms underlying VSTM for image contrast do not support “perfect storage,” as has been found for other stimulus properties (e.g., Vogels & Orban, 1986). In particular, for 2-D pixel noise patterns, discrimination thresholds increased as the memory duration increased from 0.3 to 5 s. Indeed, the increase in thresholds was substantial (about 120 % for the identical-pattern condition of Exp. 1).

More importantly, we observed a differential effect on thresholds, depending on whether discrimination was based on the same or on a contrast-reversed version of the spatial patterns. It is worth noting that everything (including the contrast polarity of each element) within the pattern was kept the same in the identical-pattern condition, and thus observers simply needed to detect the increment of contrast across the whole pattern. However, for the reversed-pattern condition, although the overall contrast energy remained the same as in the identical-pattern condition, many features of the pattern were changed by inversion. These features include the locations of dark/light and light/dark edges, as well as the absolute locations of luminance maxima and minima within the pattern. If participants used configural or local information as a reference to discriminate contrast, this would explain why the performance became much worse under these circumstances. One possible interpretation of this phenomenon is that information about the contrast to be remembered is tied in some manner to the whole pattern, such that VSTM for image contrast cannot be completely decoupled from its spatial configuration.

An alternative to coding and remembering the holistic stimulus pattern as a reference for contrast is that the visual system might only code a subset of image elements in memory. If this were the case, then observers could have performed the task by comparing the contrast between corresponding squares or clusters of elements in the comparison and test patterns. Although the stimulus in each case was a binary image of random noise, the individual luminance elements were sufficiently large to be readily resolvable, and the contrast was uniform across the image. Thus, the local contrast could be used as a proxy for the contrast of the entire stimulus, and the representation of local contrast between a few adjacent bright and dark square elements could support correct performance in the delayed-contrast discrimination. In an experiment using a delayed pattern discrimination task, Cornelissen and Greenlee (2000) found that the contributions of the elements near fixation were greater than those of elements on the perimeter of the pattern. They interpreted their data as the results of an attentional mechanism operating at the encoding stage or during the maintenance and retrieval stages. In the present experiments, it was also possible that the central regions of the display played a more important role in encoding and sustaining the representation of contrast. Our present results, however, cannot speak to this issue, so this will be an interesting question to be addressed in future research.

Experiment 1 and its results leave open the possibility that the contrast representation in the memory task is related to the activity of higher-order cognitive areas, because they are often assumed to play a role in integrating features (Pasternak & Greenlee, 2005; Ranganath, 2006; Ranganath & D’Esposito, 2005). If contrast and other pattern information can be thought of as separate features, one might argue that early sensory visual cortex is unlikely to be involved in the process of retaining information in VSTM. To address this issue, in Experiment 2, we performed a memory-masking experiment in which we delivered a mask consisting of a spatial pattern midway through the memory interval. Previous psychophysical experiments had also explored the memory process of visual features using the method of memory masking (see, e.g., Magnussen & Greenlee, 1992; Magnussen, Greenlee, Asplund, & Dyrnes, 1991). Using a similar memory-masking procedure, they measured discrimination thresholds for the spatial frequency and velocity of drifting gratings when a mask was displayed during the memory interval. Their results showed that this brief presentation of an extra, yet irrelevant, stimulus significantly increased the discrimination thresholds relative to the no-mask condition, suggesting that VSTM was affected by a mask in the same feature domain.

In Experiment 2 of the present study, the noise patterns used in the comparison and test intervals were always the same, so any additional threshold elevation must have been caused by the presence of the mask stimulus. Our results suggest that ongoing VSTM processes can be disrupted by disparate sensory input. Masking is only expected if the masker interferes at a level where contrast is processed. We found that Weber fractions increased above a no-mask baseline when a mask was present over a range of different mask contrasts, and we attribute this deterioration in performance to factors operating on low-level sensory representation. In Experiment 2, we also examined the extent to which the perception of and memory for contrast relies on an image/pictorial representation. We found that performance was most affected when the spatial configuration of the noise elements within the mask was different from, as compared to when the spatial pattern of the mask was identical to, those in the stimulus intervals.

The extent to which early sensory cortical areas also participate in VSTM is an open question. Our behavioral data suggest that a VSTM trace can be disrupted by another stimulus pattern, even though observers were not required to remember it. Despite the mask pattern being irrelevant to the task per se, it still had a disruptive effect on memory performance. Information about the global contrast and information about the spatial pattern of the stimulus appear not to be stored independently of each other for making contrast discrimination judgments, given the effects of pattern inversion (Exp. 1) and masking (Exp. 2). Therefore, visual sensory areas, where these properties are encoded jointly, may play a role in the process of VSTM for contrast.

To investigate the neural basis of VSTM for contrast, we have recently begun to use a combination of fMRI and multivariate classification analyses to look at early visual cortex (Xing, Ledgeway, McGraw, & Schluppeck, 2013, see also Ester et al., 2009; Harrison & Tong, 2009; Sneve et al., 2012). The results from these studies suggest that information about the contrast of remembered gratings is present in the fMRI responses in early visual cortex, but to what extent these signals are *causally* linked to VSTM for contrast will require further experimentation. However, regardless of the anatomical substrate that mediates VSTM, the present study suggests that the encoding of contrast in VSTM is closely tied to the spatial configuration of stimuli and is not transformed into a more abstract, feature-invariant representation.

## Footnotes

1
http://gru.brain.riken.jp/doku.php/mgl/overview
